# Noise Management by Molecular Networks

**DOI:** 10.1371/journal.pcbi.1000506

**Published:** 2009-09-18

**Authors:** Frank J. Bruggeman, Nils Blüthgen, Hans V. Westerhoff

**Affiliations:** 1 Regulatory Networks Group, Netherlands Institute for Systems Biology, Amsterdam, The Netherlands; 2 Life Sciences, Centre for Mathematics and Computer Science (CWI), Amsterdam, The Netherlands; 3 Integrative Bioinformatics, VU University, Amsterdam, The Netherlands; 4 Institute of Pathology and Institute of Theoretical Biology, Charité Universitätsmedizin Berlin, Berlin, Germany; 5 Manchester Centre for Integrative Systems Biology, University of Manchester, Manchester, United Kingdom; 6 Molecular Cell Physiology, Netherlands Institute for Systems Biology, VU University, Amsterdam, The Netherlands; University of Washington, United States of America

## Abstract

Fluctuations in the copy number of key regulatory macromolecules (“noise”) may cause physiological heterogeneity in populations of (isogenic) cells. The kinetics of processes and their wiring in molecular networks can modulate this molecular noise. Here we present a theoretical framework to study the principles of noise management by the molecular networks in living cells. The theory makes use of the natural, hierarchical organization of those networks and makes their noise management more understandable in terms of network structure. Principles governing noise management by ultrasensitive systems, signaling cascades, gene networks and feedback circuitry are discovered using this approach. For a few frequently occurring network motifs we show how they manage noise. We derive simple and intuitive equations for noise in molecule copy numbers as a determinant of physiological heterogeneity. We show how noise levels and signal sensitivity can be set independently in molecular networks, but often changes in signal sensitivity affect noise propagation. Using theory and simulations, we show that negative feedback can both enhance and reduce noise. We identify a trade-off; noise reduction in one molecular intermediate by negative feedback is at the expense of increased noise in the levels of other molecules along the feedback loop. The reactants of the processes that are strongly (cooperatively) regulated, so as to allow for negative feedback with a high strength, will display enhanced noise.

## Author Summary

Within cells, fluctuations in molecule numbers are inevitable, since the synthesis and degradation of molecules are not synchronised. Such molecular noise can be transferred to other molecules through regulatory interactions. Noise in molecular networks, and especially in gene expression, has been studied extensively over the past years, both experimentally and through mathematical modelling. In this work, we present a theoretical framework that merges concepts derived from metabolic control analysis (which was originally developed to describe the control in metabolic pathways) with linear noise approximation (a concept from statistical physics). This framework is useful to analyse how noise propagates through molecular networks, how noise can be managed within the networks and how different network designs reduce or enhance noise. The present theory makes use of the natural, hierarchical organization of regulatory networks and makes their noise management more understandable in terms of network structure. Within this paper, we apply the framework to signaling and regulatory cascades, and analyse how feedback and time scale separation influence noise propagation in molecular networks.

## Introduction

Some molecular processes involved in cellular regulation operate in a regime of low molecule numbers (a few to tens per cell). Inevitable fluctuations in reaction rates, induced by thermal noise, can then bring about significant heterogeneity in isogenic populations by causing fluctuations in copy numbers of molecules [1]–[6] (reviewed in [7]–[10]). Fluctuations arise in molecule levels because of the asynchronous occurrence of synthesis and degradation events. The extent of noise in the molecule number of a species is commonly captured by a measure of noise significance, defined as the variance in the copy number divided by the squared mean copy number, as determined from many single-cell snapshots for a population of (isogenic) cells. For molecules engaged in equilibrium reactions, this ratio is of the order of 1 divided by their mean copy number, making noise in such molecule numbers irrelevant whenever the molecule numbers exceed 100. For systems away from thermodynamic equilibrium and depending on kinetics and stoichiometry, noise can become appreciable even at high copy numbers for molecules (hundreds to thousands per cell) [11]. The noise in a specific molecule is partially determined by the molecules it forms a network with, due to noise propagation [12]–[14]. A molecule with a large copy number might display large noise, due to its communication with a molecular species with a low copy number. This may explain much of the noise found in the levels of many proteins. Despite their high mean levels, many of them are short-lived and are translated from mRNAs occurring at low levels [7],[8].

Our understanding of the functional consequences of particular network structural aspects, such as feedback, cascades, cooperative enzymes, and time-scale separation, has profited greatly from numerous theoretical studies in the last decades (e.g. [15]–[21]). Many of these studies adopted a metabolic control analysis perspective on metabolic and hierarchical networks; where the latter networks may involve signaling and gene expression. [17], [22]–[24] (recently reviewed in Bruggeman et al. [25]). They focussed on deterministic (macroscopic) network properties rather than taking a stochastic (mesoscopic) perspective. Hierarchical networks consist of modules, called levels in this framework, that are composed out of reaction networks where molecules affect the rates of processes as reactants and effectors. Inter-level interactions occur via effector interactions only; this means that the regulating molecules of one level act as activators and inhibitors of processes in another level without being consumed in the latter level. Examples of hierarchical networks are gene-expression and signaling cascades or metabolic systems involving gene expression and signaling.

Metabolic control analysis and its theoretical extensions have shown that sensitivity amplification and feedback in hierarchical networks allows for a repertoire of mechanisms for ultra- or insensitive (robust) responses to changes in particular signals and it has given us insight into distribution of control in metabolic networks [15], [16], [23], [24], [26]–[28]. These mechanisms also play a pivotal role in noise propagation through signaling and gene-expression cascades as indicated by experimental work and numerical simulations [12]–[14]. Noise transmission depends on the strength of macromolecular interactions (sensitivity) and on time scales. High frequency fluctuations in the copy number of one molecule can only transfer to other molecules if it affects an enzyme (or uncatalyzed process) that operates at even faster (internal) kinetics. If the reaction catalyzed by that enzyme involves other molecules, then noise will be transferred to the molecule numbers of these reactants if the enzyme is fast enough to track the fluctuations in the regulator. If the enzyme was not sensitive to the regulator, noise transfer would not have occurred.

Stochastic (mesoscopic) dynamics of molecular networks can be described by the so-called master equation, which models a Markov process (with continuous time and discrete-state space). It specifies the rate of change of the probability density functions for all the copy numbers of molecules (the system's state) over time [29]. Linear noise approximation (LNA) [11],[12],[29],[30] provides a first order approximation of the dynamics of the probability densities described by the master equation. It provides exact solutions for networks described by linear rate equations (e.g. of the sort, 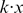 but neither 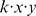 nor 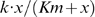).

We will reformulate LNA in terms of response analysis (RA), developed within the framework of metabolic control analysis [24],[26],[31], with the aim of merging the two methods. In this way, we can exploit the extensive knowledge about control and responses of hierarchical molecular networks within metabolic control analysis for studying the principles of noise propagation. In the first section, we introduce LNA and RA to derive the basic equation of the new, combined theory. Subsequently, we describe how noise in a single molecular intermediate is received and transmitted by its surrounding molecular network. We derive equations that indicate how noise is transmitted along cascades and how it is modulated by processes that operate at certain time-scales, feedback and feedforward loops. The analysis yields new insight into potent mechanisms for noise reduction, as well as in which mechanisms may be at the origin of the frequently observed heterogeneity of clonal cell populations.

## Results

### Derivation of the theoretical framework

The deterministic dynamics of the average copy numbers of molecular intermediates of biochemical reaction networks are often described by a system of ordinary differential equations in the following form [17] (assuming a single compartment),
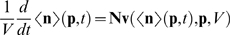
(1)


The stoichiometric matrix 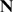 has as entries 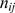, which denote the stoichiometric coefficient of the 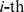 molecular intermediate in the 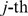 reaction. The rate vector 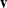 has as entries the rate equations of the reactions. The rate equations depend on the copy numbers of molecules, compartment volume (V) and kinetic parameters (entries of 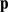). Without loss of generality, we assume that the system is described in terms of independent variables, i.e. no linear dependencies occur in the rows of 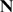 [32]. The units of 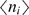 are copy numbers per cell; concentration is obtained by division by system volume (V).

In a macroscopic steady state, with steady state molecule numbers 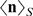 (solution to Eqn. 1 at steady-state conditions), an estimate of the magnitude of fluctuations can be obtained with linear-noise approximation (LNA) [9],[11],[29],[30]. LNA prescribes a Gaussian distribution for the probability density function of the molecular numbers at steady state. In steady-state LNA, the covariance matrix 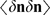 derives from the following fluctuation-dissipation theorem,
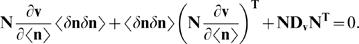
(2)


It contains the Jacobian matrix 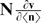, the rates 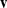 and the stoichiometric matrix 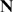. A diagonal matrix is denoted by 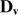, with the elements of vector 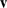 as diagonal elements. All factors of Eqn. 2 are evaluated at a (asymptotically-stable) steady-state of reference of the macroscopic system description. Since, each elementary reaction can induce noise, reversible reactions have to be split into their forward and backward elementary rate. If the units are taken to be concentrations rather than copy numbers, the volume 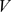 appears as a multiplier in front of the last term in Eqn. 2 [11]. LNA is commonly derived as a mesoscopic limit of the master equation, only then does the probability density function for the state become a multi-variate Gaussian distribution. Even though, LNA is strictly not applicable to processes having only a few molecules as reactants in our experience it works remarkably well in those regimes.

We shall now reformulate Eqn. 2 in terms of quantities that are used to describe responses and noise levels of molecular systems, i.e. local response coefficients and noise strengths. Control and responses of modular and hierarchical systems, such as gene-expression and signaling cascades or metabolic systems involving gene-expression and signaling, have been studied as extensions to metabolic control analysis [22]–[24] (recently reviewed in Bruggeman et al. [25]). Hierarchical networks are composed out of reaction network segments, so-called levels, that interact not by way of mass flow but solely via regulatory influences. This means that the regulator, originating from one level, where it is being synthesized and degraded, acts as a modifier of a rate in yet another level without it being consumed by the latter process. Hereby the stoichiometric matrix of the entire hierarchical network becomes block-diagonal, which provided the mathematical basis for hierarchical control analysis and modular response analysis [22]–[24]. In this work, noise transmission occurs between levels. Intra-level noise transmission can also be treated by LNA. This is not our aim here. The work of Levine and Hwa [33] considers intra-level noise propagation for metabolic networks.

Response analysis describes the responses of levels with respect to each other. Local response coefficients quantify the interaction strengths between species of different levels. Local response coefficients are given by 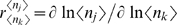 - which we shall often denote as 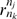. They denote the fractional change in the average steady state copy number of molecule 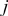, 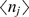, (in a recipient level) upon a fractional change of the mean copy number of molecule 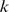, 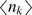, in a sender level; while keeping all other molecule copy numbers fixed at their reference steady-state values. Hence, local response coefficients quantify the strength of direct interactions between levels in hierarchical regulatory networks. The matrix of local response coefficients can be interpreted as a normalised Jacobian matrix [24],[31],
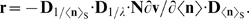
(3)


Global responses of molecular networks to changes in their environment can be understood in terms of the strength of the molecular interactions and the network structure using modular response analysis [24]. Modular response analysis derives from modular approaches to metabolic control theory. In earlier works modular response analysis was used to determine interaction strengths from steady state and transient data [31],[34].

The diagonal values 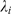 of the Jacobian matrix 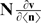 equal 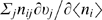 with 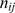 as the stoichiometric coefficient of the 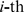 intermediate in reaction 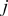. Their reciprocal values are the entries of the diagonal matrix 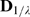. They can be interpreted as local or intrinsic eigenvalues for each variable, i.e. when all other variables are held fixed at their steady-state values. Intrinsic eigenvalues determine the intrinsic dissipation time scale of a molecular species as determined by its synthesis and degradation reaction, as one would obtain 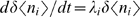 for the decay of a fluctuation in species 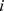 in the (artefactual) condition that all other variables were held fixed. (This does not describe the normal response of the system to a fluctuation in 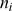; then it would bring about a response in other species which in principle could affect the dissipation of the fluctuation in 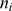 through network-level feedback.) A local eigenvalue defines an intrinsic dissipation time scale 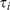 (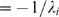) and the units of 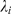 are therefore 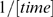. For the simple case of synthesis and degradation of 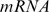, each described with mass action kinetics, 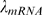 would equal the 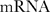 degradation rate constant, 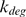. The life time of the mRNA would be given by 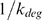.

Molecular noise is often expressed in terms of a noise strength, 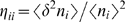, corresponding to a squared coefficient of variation. Noise strengths appear as diagonal entries in the normalised covariance matrix,
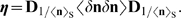
(4)


The off-diagonal entries are scaled co-variances, i.e. 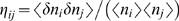. They quantify the correlations between fluctuations. If they equal ‘
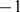
’, ‘
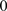
’, or ‘
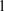
’ fluctuations in the copy numbers 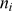 and 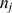 are anti-correlated, uncorrelated, and positively correlated, respectively. Reformulation of the fluctuation-dissipation theorem in terms of interaction strengths (response coefficients) and noise strengths yields the following relation,
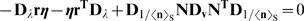
(5)


This equation merges response analysis for hierarchical networks with linear noise approximation. The term on the right is the so-called diffusion matrix which captured the fluctuation generating potential of the network. This potential increases with the stoichiometric coefficients and the rate of reactions. Its magnitude is reduced by the steady-state molecule numbers. The two terms on the left of Eqn. 5 capture the fluctuation dissipating potential of the network. This potential depends on interaction strengths and increases with a higher values for the intrinsic eigenvalues, which act as rate constants for fluctuation dissipation. Interaction strengths have a dual role, as we shall see below, they can contribute to the enhancement and reduction of noise. Since, they act also as the determinants of robustness, signal sensitivity and homeostatic properties of networks, they will prove very important in this work. Even though changes in their values may be beneficial to signal transmission, they may at the same time enhance noise propagation. We will show how such negative side effects can be modulated in networks by time scale separation and feedback design.

Below we will outline a method where each molecule is considered as a noise source in a hierarchical network. All noise generated by processes somewhere in the system propagates through the hierarchical network via the direct interactions, paths and cycles between network segments that act as levels. The strengths of these interactions are captured in terms of local response coefficients and enhance or reduce the resultant global noise in the level of molecular species. Noise propagation will be shown to depend on the amplifying or attenuating potential of molecular interactions and the time scale of interaction paths in the network. LNA is not restricted to hierarchical networks, noise transfer between molecules that are linked via stoichiometrically coupled interactions can be treated as well. Here we report only the analysis of hierarchical networks.

### Intrinsic noise: single molecular species as noise sources

**Figure 1:**
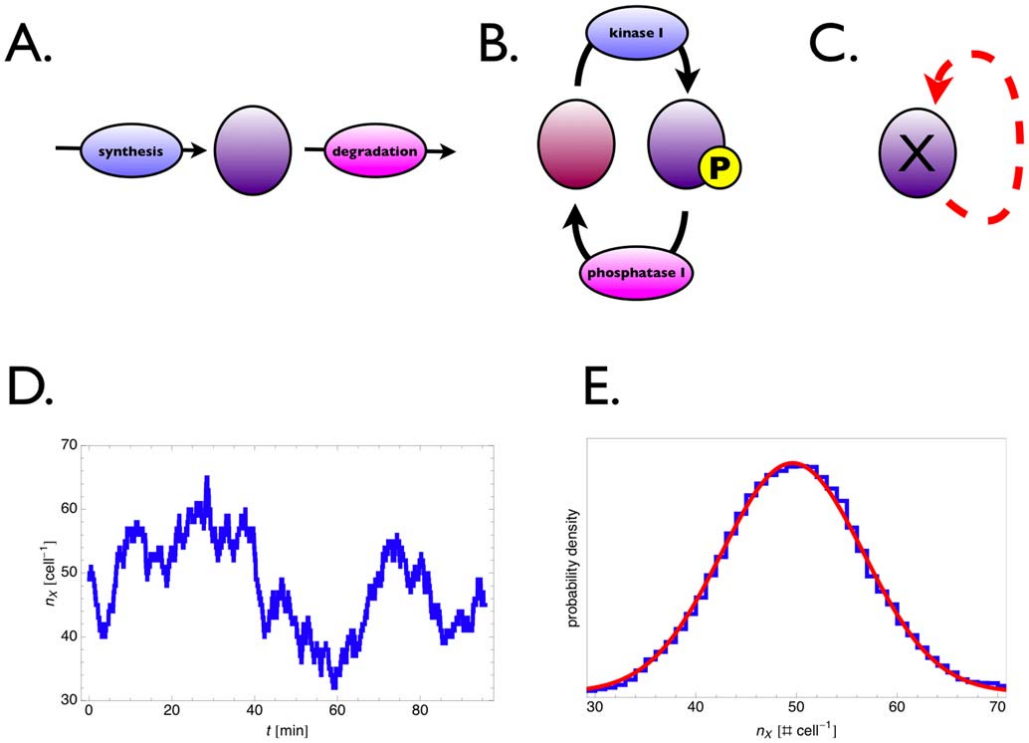
Two molecular networks with a single (independent) molecular intermediate and illustration of molecule noise. (A) A synthesis and degradation network and (B) a covalent-modification cycle (middle). Each of these networks can be depicted as a self-regulating intrinsic noise source (C), which acts as a noise transmitter in large networks. A more complicated network that still qualifies as a valid intrinsic noise source would be a molecule having multiple synthesis and degradation reactions. (D) A representative steady-state trajectory for a molecule copy number per cell, e.g. a mRNA. In (E), the steady state copy number distribution is displayed; analytically as a Gaussian distribution (red line) and from stochastic simulations with the Gillespie algorithm (blue line). The Gaussian distribution is the LNA estimate, with the mean deriving from the macroscopic description (Eqn. 1) at steady state and the variance from Eqn. 2.

We illustrate how noise arises in a molecular network by considering a simple system first; one molecular intermediate is converted by a single synthesis and degradation reaction. A generic network is depicted in Figure 1C, A and B show specific examples. Hereby we gain insight into how noise is generated in larger networks, which we will treat in the following sections.

Intrinsic noise in the copy number of a molecule is the noise that remains when all other molecules in the network are held constant. Application of the above derived formalism yields the following expression for the intrinsic noise strength (cf. [9],[11]):
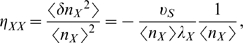
(6)


The net steady-state flux through the system is denoted by 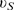. The first factor in Eqn. 6 equals the concentration control coefficient of the synthesis rate; denoted by 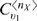 in terms of metabolic control analysis. This coefficient quantifies the extent of control of the synthesis reaction on the steady-state copy number of molecule 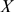, 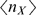, as the fractional response in this amount upon a fractional change in the activity of the synthesis process (e.g. 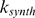). In the simplest pathway design, with the first reaction product insensitive and the second reaction first-order in 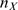, this control coefficient equals 1. Then, the noise equals that of the Poisson distribution obtained for systems at thermodynamic equilibrium, i.e. 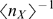. Indeed this control coefficient, which equals minus that of the second reaction on the concentration of 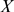, measures the extent to which the copy number noise deviates from this classical picture. If the first reaction is product insensitive and the second reaction saturated with X, then the control coefficient can become quite high and the noise can much exceed the Poisson case. In more complicated situations the sensitivities of both reactions are variable. For a general biochemical pathway (Figure 1A), the intrinsic eigenvalue, containing the sensitivities (elasticity coefficients), equals, 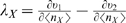. In this situation, the noise depends on the state of the network.

In a covalent-modification cycle (the signalling system depicted in Figure 1B), the intrinsic eigenvalue is given by 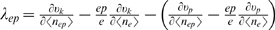; with 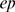 as the phosphorylated form of the enzyme (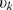 and 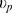 denote the rates of the kinase and the phosphatase, respectively). When this cycle operates in its ultra-sensitive regime [27] it will display large noise.

It is illuminating to interpret Eqn. 6 in terms of the timescales in the system. The time scale of the generation of fluctuations is given by the turnover time of 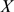, 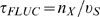 ([time/(generated fluctuation of size 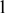 molecule]). The ‘local’ eigenvalue 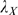, which corresponds to a diagonal element of the Jacobian matrix, provides an estimate for the timescale to dissipate fluctuations in 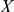, 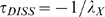 (unit: [time/(dissipated fluctuation of size 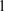 molecule)]). The ratio of these times gives the accumulated size of the fluctuation during the time required to dissipate a fluctuation of size one molecule. Rewriting equation (6) gives,
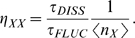
(7)


This equation indicates that if the time to generate a fluctuation would be increased, such that a smaller number of fluctuations are generated per unit time, the noise would reduce (at a constant dissipation time for a fluctuation). In other words, the accumulated deviation from the average number of molecules - the noise - would be reduced. Similarly, a reduction in the dissipation time for a fluctuation would also reduce the noise.

Equation 6 provides an exact solution of the master equation if the rate equations for the decay process(-es) is (are) linear in the copy number of 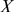, and the production is independent of 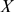. Under such conditions, the noise equals 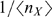. In case of nonlinear rate equations (mass-action or elementary complex (Michaelis-Menten)), LNA becomes an approximation. Noise then depends in addition on rate sensitivity coefficients (so-called elasticity coefficients), which determine 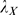.

The inverse of the mean copy number of a molecular intermediate, which is often taken as a (Poissonian) noise estimate, has only limited validity in molecular networks. It applies, for instance to the network: 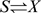 with 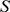 fixed, i.e. at thermodynamic equilibrium, and 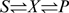 with 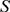 and 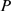 fixed, i.e. at steady state, under the condition that the reactions are described with first-order mass action kinetics. If 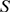 and 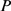 are assumed variable, the noise equals 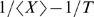, with 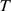 as 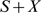 or 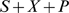. For signaling cycles or linear pathways, operating at their ‘ultra-sensitivity’ regime, i.e. large 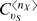 in Eqn. 6, the intrinsic noise can be much higher than the Poissonian estimate.

In this work, the network displayed in Figure 1 will be considered as an generic noise source. In the sections that follow we will consider how its noise propagates through hierarchical networks and under what conditions it may be enhanced or attenuated in specific network designs. Levine and Hwa [33] took an orthogonal perspective to ours and increased the complexity of this network in an intra-level fashion. They considered noise propagation in metabolic networks rather than hierarchical networks. They found evidence for little noise propagation between metabolic intermediates for flux-driven metabolic pathways with enzymes having little or no sensitivity to the concentration of their product(-s). Such enzymes have been called slave enzymes in metabolic control analysis [35].

### Noise propagation in dictatorial hierarchical networks

**Figure 2:**
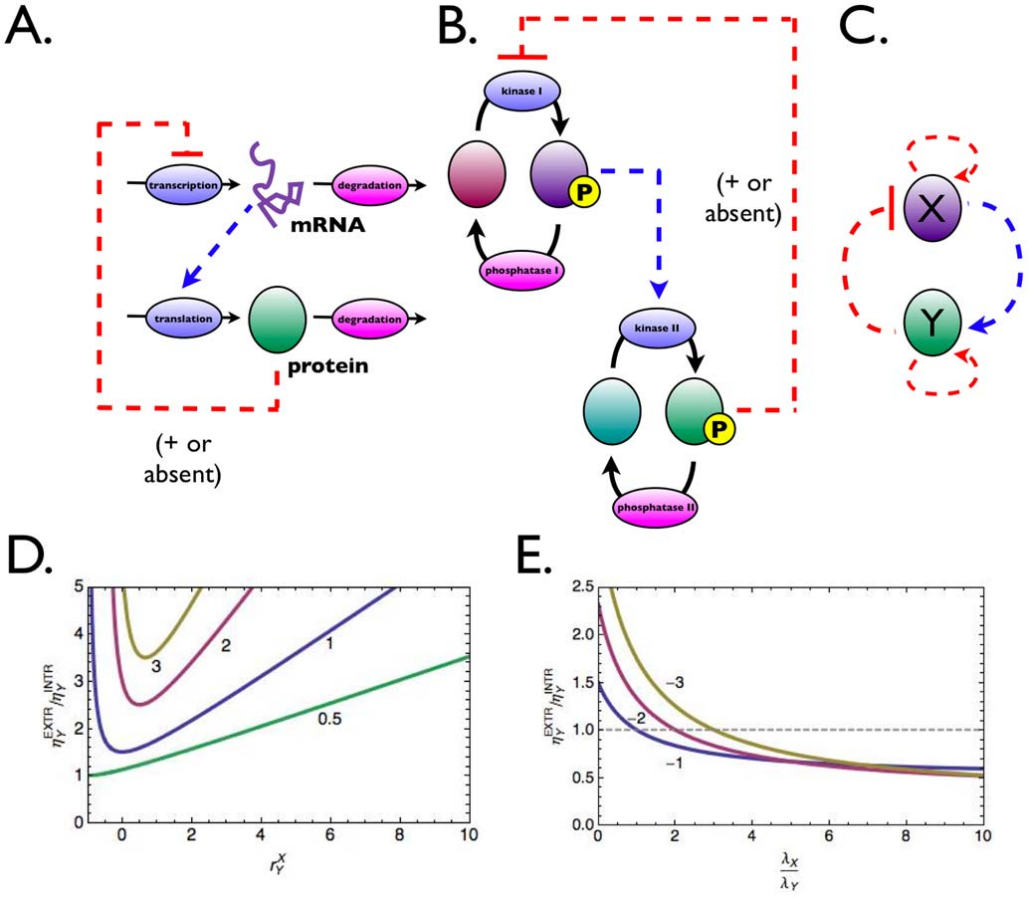
Two-level cascades with feedback regulation. A transcription-translation (A) and signal transduction two-level network (B), each can be reduced to the generic scheme shown in (C), using the model reduction explained in Figure 1. Figure D shows that ultrasensitive system responses, i.e. 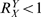, (the 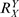 value is indicated by the numbers in the plot) for the network displayed in (C) are accompanied by minimal noise in case of positive feedback regulation (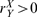). (E) Time scale separation can reduce noise in the network displayed in (C) in the case of negative feedback, (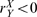, its values are indicated as numbers).

In this section, we are interested in determining how the intrinsic noise of a molecule 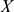 propagates to a second molecule 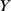, e.g. from mRNA to protein (Figure 2A) or from a kinase to its target protein in a signal transduction cascade (Figure 2B). We consider that solely the synthesis of 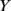 is regulated by 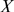 (Figure 2C, with the feedback of 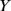 onto 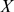 absent). For simplicity, we assume that 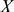 is not regulated by any other species and therefore its net noise is captured by Eqn. 6. In the next section, we will consider feedback. Within the formalisms of control and response analysis, the resulting network resembles a dictatorial hierarchical network composed out of two levels with mass flow occurring solely within these levels [25]. The levels are coupled by way of the regulatory effect of 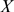 on the synthesis rate of 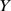; 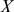 is not consumed in this process but acts solely as an effector.

The analytical solution of the covariance matrix from the FDT relationship (Eqn. 5) indicates that the network-level noise (or global noise), 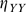, in the level 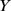 at steady state equals the sum of two terms,
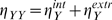
(8)


In this relationship, 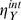 represents the intrinsic noise in 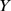 analogous to the noise for 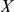 as given in equation (6). The second term in Eqn. (8) expresses the extrinsic noise in 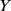, noise that originates from a molecular species converted in another level than the one where 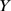 is inter-converted. If stoichiometrically-coupled molecules are considered, the noise would originate from a molecule which is one reactants of a reaction involving 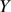.

The extrinsic noise is composed of a multiplication of three factors; (i) the squared sensitivity of 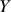 to 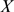, captured by the local response coefficient, 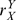, (ii) the time scale separation between 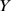 and 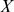, and (iii) the intrinsic noise in 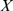,
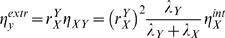
(9)


The 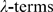 in this equation should be interpreted as first-order rate constants for the dissipation of fluctuations. Alternatively, the time scale separation term could have been written in terms of characteristic life times for fluctuations in 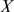 and 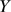 as, 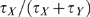 (with 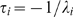).

Eqn. 9 indicates that the extrinsic noise is always positive. It does not matter whether the effect of 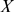 on the synthesis of 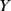 is stimulatory or inhibitory. This indicates that the global noise in 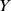, 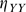, can not be reduced below 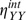 by having an external controlling level (mediated by 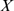) in such a cascade. Thus, 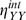 is the minimal noise in 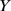. This limit is attained if the fluctuations of 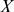 decay much faster than those of 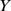: 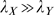; then 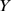 can only track the mean of 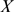 rather than its fluctuations. As we shall see below, negative feedback between the levels of 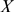 and 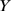 can reduce noise below 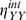.

Eqn. (8) also shows another interesting effect. Even at a high average level of 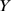, such that its intrinsic noise is low, its global noise can be high nonetheless as a result of noise propagation. The (global) noise in 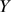 is then dictated by the noise in the intermediate of its controlling level, i.e. in 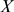. For instance, because 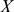 occurs as a low copy number molecule. Alternatively, the noise in 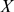 can be amplified, i.e. when the reaction (at the level of 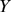) that is directly sensitive to 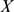 has a high control coefficient on the steady-state copy number of 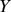. A high control coefficient is not a necessary condition for significant noise propagation as it still depends on the time scale separation between 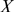 and 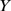; if 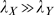, noise propagation is reduced.

In the linear hierarchical network treated above, extrinsic noise was shown to be equal to 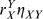 (Eqn. 9). When 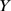 is controlled by multiple factors, extrinsic noise is given by the sum over the covariance terms with all controlling factors multiplied with the response coefficient,
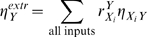
(10)


The covariance factors 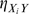 contain local response coefficients, time-scales and an intrinsic noise term (cf. Eqn. 9). In this way, “noisy” parameter influences can be introduced into linear noise approximation. Swain et al. [36] derived a more general approach to extrinsic noise in gene expression. Recently, Rocco [37] has extended metabolic control analysis to incorporate the effect of fluctuating parameters on the summation and connectivity theorems for control coefficients. A more general approach to parameter sensitivity analysis of stochastic systems (with a discrete state space) was carried out by Plyasunov and Arkin [38]. They have developed an approach that can be embedded straightforwardly in the Gillespie algorithm. Equation 10 assumes that the external noise of different sources is completely uncorrelated. If this is not the case, but the noise is for instance generated by a network with unknown dynamics then this system may force the network of interest to display emergent dynamics [39]–[41].

### The influence of feedback on noise propagation

The previous section established that noise in molecule copy numbers is modulated by other network components through noise transmission. How is noise influenced by feedback between levels? One would expect that feedback introduces two consequences for noise propagation. It causes the intrinsic noise of a particular molecule, say 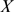, to loop through the network to return to 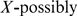 via multiple loops each having different molecular components and time scales. In addition, all the other molecules that 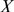 receives information from will act as noise sources transmitting noise to 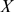. Inspired by this intuition, much of the general effect of feedback on noise can be understood using a simple extension of the model we considered above. More complicated cases will be considered in subsequent sections.

We extend the network treated in the previous section with a regulatory effect of 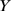 onto either the producing or consuming reaction of 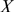 (Figure 2C). The net effect is the appearance of a new interaction quantified by the local response coefficient 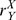. The extrinsic noise term of 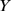 changes from Eqn. 9 into (its intrinsic noise remains unaltered, see Eqn. (6)),
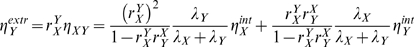
(11)


The first term captures the transmission of noise from 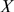 to 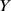 modulated by the feedback loop (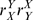), which occurs in the denominator. The second term describes the noise reverberation along the feedback loop, e.g. the attenuation or amplification (depending on the sign of the feedback loop) of intrinsic noise of 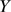 through the feedback. The strength of the feedback is given by 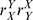. If the interaction from 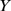 onto the level of 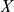 is removed, i.e. 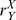 is set to zero, this equation reduces to Eqn. 9.

In case of positive feedback, the feedback strength 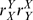 is limited to values below 1 as otherwise a saddle-node bifurcation occurs. Positive feedback always increases noise above intrinsic noise alone, as both terms are positive in Eqn. 11. When we consider the following simplification: 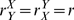, 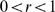 and 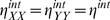, 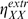 is given by
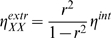
(12)


Under this condition, global noise terms would simplify to 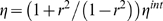. If 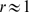 the noise becomes much higher than 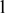. It can be shown that this condition coincides with the determinant of the Jacobian matrix to become zero, which indicates that the system operates close to a saddle node bifurcation. In the next section, we will consider negative feedback.

### Negative feedback: conditions for noise reduction and a trade off

If feedback is negative, the first term on the right-hand side of Eqn. 11 is positive and the second term negative. Noise is reduced by the extrinsic factor, through the feedback loop, provided the second term dominates in magnitude. This is the case under the following conditions for the time scales: 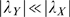, i.e. the time scale of the dynamics of 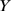, should be much longer than for the dynamics of 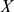. Consequently, 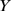 responds too slowly to be able to track the fluctuations in 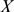. If this is not the case, i.e. when 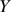 responds faster than 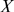, the first term dominates and negative feedback enhances the noise of 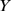. This is shown numerically in Figure 2E. Under those conditions, the opposite phenomenon occurs: the noise in 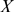 will now be small; as 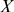 now responds too slowly and can not track the fluctuations in 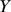
! Interestingly, these conditions show that integral feedback controllers might not be optimal if low numbers of molecules are involved. Since integral feedback controllers require feedback with slow dynamics they will bring about large noise. In other words, reduction of noise in one molecule through a feedback loop through another molecule will increase the noise in the latter molecule.

An additional possibility to reduce noise arises when the feedback is strong, but the feedback strength is not equally distributed: 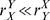, i.e. when 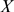 responds very sensitively to 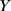, but 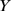 only weakly to 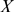. Under these conditions the second term may become large. This result points to a possible design for noise reduction: the negative feedback should be such that an allosteric interaction should run from 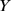 onto the synthesis or degradation reaction of 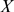 and not vice versa if the noise in 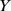 is to be reduced by feedback. This may contribute to noise reduction at the protein level as translation depends linearly on mRNA levels whereas transcription can depend on transcription factor concentrations in a strongly nonlinear fashion.

The extrinsic noise equation for 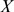 is the symmetrical counterpart of Eqn. 11. Noise reduction in 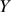 occurs if 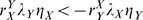 then the second factor in Eqn. 11 dominates. This condition is exactly the condition for noise increase in 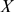
! Thus, there exists a trade off: the noise reduction in 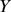 occurs at the expense of a noise increase in 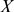.

### Optimal positive feedback design for ultrasensitivity

The terms, 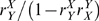 and 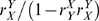, in equation (11) are examples of internal global response coefficients, respectively denoted by the global response of 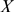 upon a change in 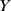, 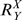, and vice versa, 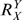. These are central expressions in modular response analysis and portray network-level responses [24],[26]. Each gives a systemic change in the steady-state value of an output with respect to a perturbation in another state variable, which can be expressed in terms of strengths of interactions between state variables. The resulting expressions always contain strengths of interaction paths and loops in the network, such as 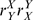 [24].

The relation between global response coefficients and noise propagation analysis can be used to understand trade-offs between the responsiveness of a network, either at the network-level or at the level of single interactions, and its noise characteristics. Hornung and Barkai [42] recently reported that responsive networks have reduced noise if they are controlled by a positive feedback. This counter-intuitive observation can be understood using the present framework. Substituting the global response coefficient in Eqn. 11 yields:
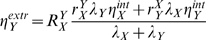
(13)


In order to yield a positive global response coefficients, the cascade amplification 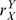 needs to be positive. The strength of cascade amplification for a given global response coefficient 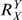 and a given feedback strength can be determined by:
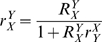
(14)


Therefore, extrinsic noise in Y is given by:
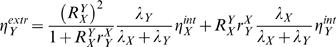
(15)


If timescales and copy numbers of Y and X are equal, for all ultrasensitive systems, i.e. where 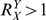, lowest noise can be obtained by positive feedback, i.e. 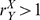. Examples are displayed in Figure 2D. However, note that the resulting network-level noise under those conditions is still larger than the intrinsic noise alone. Therefore, negative feedback is a much more potent noise attenuator for systems not requiring highly sensitive signal transmission.

### Noise transmission in a three-level cascade with and without feedback

**Figure 3:**
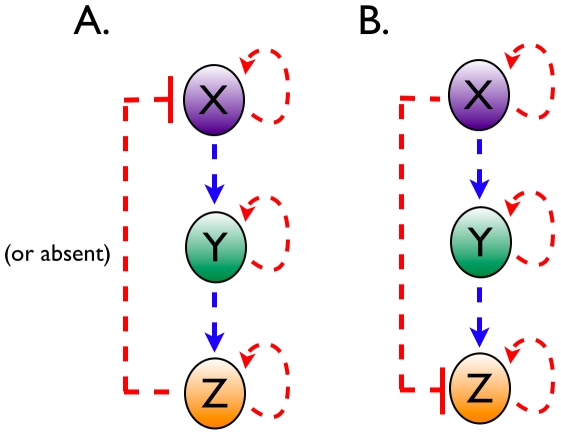
A three level cascade with a feedback and a feedforward loop. Feed-back (A) and feed-forward (B) regulation occur frequently in signaling networks, and in metabolic regulation through changes in enzyme induced by altered transcriptional and translational activities.

Three-level cascade networks arise often in molecular networks, e.g. in signaling (e.g. MAPK) and gene networks [43] (Figure 3). Cascade design is the basal organization of hierarchical networks involving transcription, translation and protein-function networks. We shall now extend the two-level cascade design analyzed in the previous section to a three-level design. The noise in the level of the output intermediate 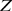 of a linear three-level cascade without feedback is given by,
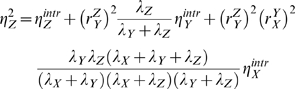
(16)


Comparison with Eqn. 9 shows that an additional term appears when a third level is introduced. This term (the last) captures the noise transmission from 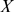 to 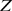 as relayed by 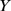. This term would be negligible if: (i) 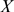 would be held fixed, (ii) 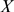 would be a very fast responding variable, i.e. if 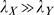 and 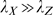, or (iii) when 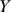 is insensitive to 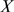, 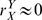. The time scaling term converges to 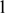 if 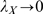 (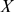 is a slow responder) and to 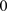 if 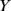 or 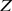 would be a slow responder - they can not track the noise in their input.

Ultra-sensitive interactions between communicating levels, i.e. 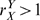 and 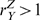, tend to increase the noise transmission along the cascade. This effect can be counteracted by time-scale separation between the levels; noise reduction occurs if 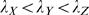. Again this corresponds to intuition. Insightful and theoretical analysis of such systems for varying cascade lengths has been carried out by Thattai & Van Oudenaarden [44]. The experimental analysis of Pedraza & Van Oudenaarden [14] provides a seminal example of noise propagation in cascades.

Equation 16 shows another interesting aspect of noise propagation in hierarchical networks: noise and signal sensitivity can be tuned independently. Multiplication of the rate equations of synthesis and degradation of 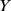 with a factor 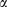 would lead to an proportional change in 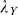 and the steady state flux through 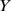. The steady state level of 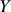, 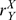 and 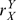 would remain unchanged. This indicates that the cascade response 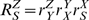 would be unaffected by such a change in the time scale of 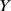. An increase in the time scale of 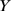 would however affect noise transmission along the cascade. Therefore, molecular networks can evolve signal sensitivity and transmission independently of noise management. This result is independent of the presence of feedback loops (see below).

We will now incorporate a negative feedback from 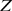 onto the synthesis term of 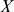. The response coefficient of 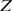 with respect to 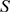 becomes in this case,
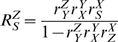
(17)


The effect of the complete feedback loop is through the denominator term. The strength of the feedback loop is captured by the product of local response coefficients, 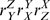. We will now illustrate with numerical simulations that the noise propagation can be affected qualitatively by the effect of the feedback loop as well as by the time scale separation with the cascade whilst the response coefficient 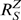 is invariant. The results are summarized in Table 1. They indicate that negative feedback can enhance or reduce noise depending on the extent of time scale separation. When the feedback is faster than 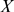, the noise in 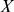 is lowest as it can track the fluctuations in its regulator 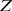. As 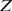 is now the fastest responding molecule it will track the fluctuations in the level of 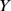 and become noisy. 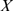 has most noise when the feedback loop is slow. In other words, the reduction of noise in one intermediate through a negative feedback increases the noise of the faster intermediates in the feedback loop.

**Table 1:** Simulations of the influences of negative feedback regulation and time scale separation on noise in the intermediates of a three-level cascade.

Negative feedback	Time scale of Y & Z	Noise (X/Y/Z)	Explanation
absent	same as X	0.25/0.38/0.47	noise propagation
present	same as X	0.34/0.34/0.34	symmetric case
present	faster than X	0.11/0.28/0.46	feedback corrects noise in X
present	slower than X	0.40/0.37/0.14	feedback corrects noise in Z

Faster (or slower) than X indicates that the synthesis and degradation rate constants of Y and Z where 10 and 100 times higher (or lower) than those of X, respectively. For all steady states, all molecules have the same copy number, and fluxes. The sensitivities (local response coefficients) do not depend on the chosen time scales for 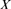, 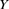, and 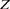 (see main text). The kinetic descriptions follow mass action, e.g. 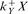 and 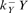 for the synthesis and degradation of 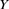, resp., except for the synthesis of 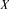, which was modelled as 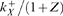. The statistics derive from at least 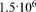 steps in the Gillespie algorithm.

If the dynamics of 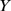 and 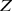 are much faster than that of 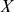, they can be considered at a quasi-steady state relative to 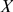. In this limit, the system dynamics can be captured solely in terms of 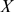. In this reduced model, 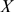 inhibits its own synthesis directly; no additional noise is introduced by 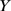 and 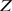 and the full potential of negative feedback as noise corrector for 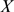 becomes apparent. In this quasi-steady state limit, the minimal noise in 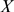 for this network parametrization corresponds to (compare to Eqn. 6),
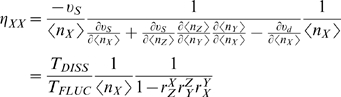
(18)


The last factor in this equation captures the reduction of noise in 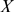 by the fast negative feedback. It is positive for negative feedback and reduces the noise more for stronger feedback. In this parameter regime, 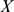 is also robust with respect to parameter changes (e.g. when parameter 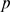 directly effects the level of 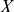, 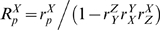 is small). Given the kinetic parameters of the subsystem for 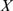, the fast feedback exerting its influence through its feedback strength (gain) 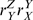, can now be designed to have a high enough gain to act as noise corrector. These results should be interpreted with some caution as they may seem to imply that zero noise is possible. Using less coarse-grained descriptions, it can be shown that diffusion and information theory set fundamental limits to minimal noise levels ([45],[46])

The incorporation of a feedforward loop in the three-level cascade affects the noise transmission in yet another manner. Such a design is shown in Figure 3. The noise in the copy number of the output of this system is given by,
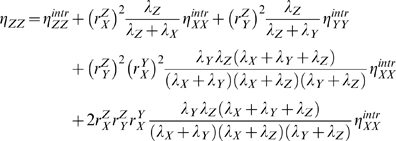
(19)


In this equation, the final term has an interpretation we have not encountered yet. It captures the synergistic effect of the two paths that run from 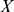 to 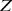. The net response coefficients of these paths, i.e. 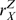 and 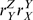, appear as products. In case of a negative feedforward loop the final term becomes negative, which would reduce the noise in 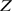.

## Discussion

In this paper, we presented a conceptual and mathematical framework that gives insight into noise management by molecular networks. Intrinsic noise in the copy number of a molecule was shown to derive from the fluctuations in the birth (synthesis) and death (degradation) rates of that molecule. The noise that a molecule exhibits in a network equals the sum of its intrinsic noise and an additional extrinsic noise component. The extrinsic noise component arises from molecular networking. Modular response analysis and hierarchical control analysis exploit the hierarchical design of most signaling networks and of transcription and translation cascades and are each extensions of metabolic control analysis [22]–[24],[26],[31]. This work presented a merger of noise and response analysis. We have focussed solely on hierarchical networks composed out of levels even though the methods outlined in this paper can be straightforwardly generalized to non-hierarchical networks.

Where on the one hand our methodology is innovative because of its tight link with metabolic and hierarchical control analysis it is similar, on the other hand, to the approaches developed by Paulson [9],[12]. The two approaches both derive from linear noise approximation (LNA) as an approach to estimate noise in molecular networks. Paulsson's reformulation of LNA offers a description in terms of concepts that draw on analogies from physics whereas we take a more control-centric perspective. Our approach makes many of the results within metabolic control analysis, e.g. dealing with cascades, feedback, ultrasensitivity, and robustness, applicable to the analysis of noise propagation. Another such link with control theory is apparent in the frequency domain approach to the analysis of noise [47],[48] and control [49].

Negative autoregulation (NAR; Figure 2) accelerates the response of small gene networks, e.g. through a transcription regulator inhibiting it's own transcription [43]. For the 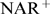 and 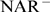 motifs to have the same steady state flux a higher synthesis rate in 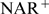 cells is needed to compensate for the inhibition by the negative feedback at steady state. The consequential reduction in time scale enables a faster dissipation of fluctuations and makes this network design more noise resistant (evident from Eqs. 6 & 11, and discussed in the accompanying sections). The noise of NAR motifs has been analyzed experimentally using synthetic gene circuits [3], [13], [50]–[52].

Besides NAR other mechanisms have been shown to reduce noise levels, i.e. dimerization of transcription factors [53], polycistronic mRNA [54], regulated protein degradation [55], and DNA looping [56]. Swain studied two variants of negative autoregulation in transcription and translation and showed that post-transcriptional regulation is a more potent noise reducing mechanism than post-translational regulation [54]. These studies are typically theoretical studies and experiments have yet to be performed to investigate whether these mechanisms influence noise management in particular cases and to significant extents.

Some of these proposed mechanisms for noise reduction rely on stoichiometric constraints besides regulatory influences. The approach discussed in this work only considered regulatory influences. When comparing the hierarchical system in Figure 2 with the network 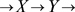 where 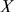 and 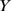 have a stoichiometric and regulatory coupling and taking the kinetics the same, i.e. for the hierarchical case the rate of 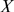 degradation and 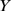 synthesis both equal 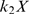, the difference between the noise in 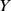 between the two systems corresponds to 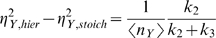. This indicates that the reduced correlation between molecule copy numbers in hierarchical networks, due to the absence of stoichiometric relations, increases noise.

Levine and Hwa [33] have considered noise in metabolic networks where the coupling between molecules is via mass flow and, in addition, possibly (allosteric) effector interactions. They found for metabolic networks driven by a product-independent flux, a pump, and composed out of enzymes, which are only sensitive to their substrate concentration, that the noise in a metabolite is independent of all other metabolites. They found that this result is fairly robust to alterations in pathway design and enzyme kinetics. This result is related to the concept of slave enzyme as defined in metabolic control analysis. Enzymes that are only sensitive to their substrates have been termed slave enzymes [35]. The steady-state concentration of any metabolite in a linear pathway composed out slave enzyme is then only determined by the pump speed and the kinetic properties of the consuming enzyme, irregardless of the number of enzymes in the pathway [35]. Changes in their concentrations can then only be brought about by a change in the pump speed or consuming enzyme level. Levine and Hwa [33] showed that the noise in a slave metabolite levels is also robust to the properties of other enzymes except for those of the consuming enzyme. How noise in enzyme levels brings about noise in metabolic flux is largely unexplored. We think that this is an important topic perhaps more important than noise in metabolite levels as they are typically large. Noise in metabolism is then much more likely to occur through noise in protein levels as their copy numbers tend to be smaller than metabolite levels and they can suffer from bursts [57].

Many experiments have shown the occurrence of transcription bursts [5], [57]–[61]. In prokaryotes, these have been shown to enhance adaptation potential [59]. Occasional fluctuations in the binding of repressors at the operators of repressed operons have been shown to cause bursty mRNA synthesis [57],[59],[60]. Hereby, some cells within an isogenic population have an adaptive advantage if the corresponding environmental change occurs purely by chance [59],[62]. The origin of bursts in eukaryotic transcription is most likely different and related to an interplay between transcription factor, and chromatin remodeling dynamics [58],[61]. LNA has been extended to incorporate bursts [5],[63] and indicates that bursts strongly enhance noise. Singh and Hespanha [64] were able to express the noise in protein level as function of the burst size and its variance. They show that noise increases with increasing burst and analyzed under which conditions noise can be reduced through auto-regulatory negative feedback (see also Friedman, Cai and Xie [65]). They find that negative feedback can both enhance and reduce noise. When transcription occurs in bursts, the waiting times for consecutive mRNAs become non-exponentially distributed and even doubly exponential [60],[66],[67]. A general stochastic theory for molecular networks that incorporates bursts and birth and death processes having non-exponential waiting time distributions is currently lacking. Such a theory, should offer deeper understanding of the constraints imposed by the stochastic nature of single cells as well as of potential benefits. At present, approximate stochastic theories, such as the one presented in this work, apply to Markov systems where all events are assumed to have a memoryless (exponential) waiting time distribution. This Markov assumption can be valid even if processes have non-exponential waiting distributions provided they do not function in synchrony and many process copies function simultaneously [67]. On the other hand, phenomena such as epigenetics, and cell heterogeneity that is inheritable, without us knowing of the determining molecular factor, suggests that extensions of the theory to non-Markovian situations might be useful.

## Materials and Methods

All calculations were performed using Mathematica. Notebooks of the calculations are available as supplementary material: Protocol S1 contains calculations for Fig. 1 D+E, Protocol S2 contains calculations for Figure 2D, Protocol S3 contains calculations for Figure 2E, and Protocol S4 contains calculations for Table 1.

## Supporting Information

Protocol S1 Calculations for Figure 1
(0.30 MB GZ)

Protocol S2 Calculations for Figure 2D
(0.01 MB GZ)

Protocol S3 Calculations for Figure 2E
(0.01 MB GZ)

Protocol S4 Calculations for Table 1
(0.12 MB GZ)
